# Fermented but Not Irradiated Cottonseed Meal Has the Potential to Partially Substitute Soybean Meal in Broiler Chickens

**DOI:** 10.3390/ani14192797

**Published:** 2024-09-27

**Authors:** Amin Ashayerizadeh, Vahid Jazi, Fatemeh Sharifi, Majid Toghyani, Hossein Mohebodini, In Ho Kim, Eugeni Roura

**Affiliations:** 1 Department of Animal Science, School of Agriculture, Shiraz University, Shiraz 71441-65186, Iran; amin.ashayerizadeh@yahoo.com; 2 School of Agriculture and Food Sustainability, The University of Queensland, Gatton Campus, Gatton, QLD 4343, Australia; v.jazi@uq.edu.au; 3 Central Queensland Innovation and Research Precinct (CQIRP), Institute for Future Farming Systems, Central Queensland University, Rockhampton, QLD 4701, Australia; fatemeh.sharifi@cqumail.com; 4 Department of Animal Science, Isfahan (Khorasgan) Branch, Islamic Azad University, Isfahan 81551-39999, Iran; toghiani@hotmail.com; 5 Department of Animal Sciences, University of Mohaghegh Ardabili, Ardabil 56199-11367, Iran; mohebodini@yahoo.com; 6 Department of Animal Biotechnology, Dankook University, Cheonan 330-714, Choongnam, Republic of Korea; 7 Centre for Nutrition and Food Sciences, Queensland Alliance for Agriculture and Food Innovation, The University of Queensland, Brisbane, QLD 4072, Australia; e.roura@uq.edu.au

**Keywords:** electron beam-irradiated cottonseed meal, fermentation, gossypol, broiler chickens, nutrient digestibility

## Abstract

**Simple Summary:**

This study examines the impact of replacing 50% of soybean meal (SBM) with untreated cottonseed meal (CSM), fermented CSM (FCSM), or electron beam-irradiated CSM (ICSM) on the growth performance and physiological characteristics of broiler chickens. The findings revealed that broilers fed ICSM achieved higher body weight than those fed CSM. Furthermore, substituting 50% of SBM with FCSM significantly improved growth performance and nutrient digestibility compared to CSM and ICSM diets. In addition, including FCSM in the diet enhanced digestive enzyme activity, balanced the microbiota population, and reduced ammonia emissions compared to other experimental diets. Overall, FCSM provided superior nutritional benefits compared to both untreated and irradiated CSM in broiler diets. Therefore, FCSM can be used as a suitable alternative to SBM in broiler chicken diets.

**Abstract:**

This study was conducted to investigate and compare the effects of substituting soybean meal (SBM) with untreated cottonseed meal (CSM), fermented CSM (FCSM), or electron beam-irradiated CSM (ICSM) on the growth performance, cecal microbiota, digestive enzyme activity, apparent ileal digestibility (AID), and excreta gas emission of broiler chickens. A total of 384 one-day-old male broiler chickens were randomly assigned to four experimental diets, with eight replicates per diet and 12 birds per replicate, for six weeks. The experimental diets consisted of a control diet based on corn–SBM and three other diets in which 50% of the SBM (control) was substituted with CSM in its raw, irradiated, and fermented forms. The results showed that throughout the entire rearing period, feeding broiler chickens with ICSM significantly increased average daily gain (ADG) and body weight (BW) compared to the CSM diet (*p* < 0.05). Replacing 50% of SBM with FCSM led to a significant improvement in BW, ADG, and feed conversion ratio (FCR) compared to the CSM and ICSM diets (*p* < 0.05). Interestingly, no significant differences in BW, ADG, or FCR were observed between birds fed FCSM and those on the control diet (*p* > 0.05). Birds fed FCSM diets exhibited the lowest pH value in the crop, ileum, and ceca. Substituting SBM with FCSM significantly reduced *Escherichia coli* and *Clostridium* spp. counts in the ceca, while enhancing the presence of *Lactobacillus* spp. (*p* < 0.05). The AID of protein and ether extract was higher in the FCSM group than in the CSM and ICSM groups (*p* < 0.05). Compared to the CSM diet, ICSM feeding improved protein digestibility (*p* < 0.05). Broiler chickens on the FCSM diet exhibited higher intestinal amylase and protease activity than those on the other diets (*p* < 0.05). Furthermore, feeding diets containing FCSM significantly reduced ammonia emissions compared to the other diets (*p* < 0.05). Overall, our results indicated that microbial fermentation of CSM is a more effective approach than irradiation for enhancing the nutritional value of CSM. Therefore, FCSM is recommended as a viable alternative protein source that can safely replace up to 50% of SBM in broiler chicken diets, particularly during times of fluctuating SBM prices and availability issues.

## 1. Introduction

At a global level, the poultry industry’s growth faces challenges due to persistent fluctuations in the cost and supply of conventional feed ingredients, highlighting the need for sustainable alternatives. Soybean meal (SBM) is the most extensively used plant-based protein source in poultry diets, owing to its high crude protein and amino acid composition [1,2]. However, the growing demand for SBM, price volatility, and competition with human food production have driven researchers and nutritionists to investigate potential substitutes for SBM in poultry diets [2,3]. Cottonseed meal (CSM), a by-product of cottonseed oil extraction, presents considerable potential as a protein source for poultry feed due to its high protein content, balanced amino acid profile, and widespread availability [1,3,4]. A key limitation in utilizing CSM in poultry feed is the anti-nutritional effects of free gossypol (FG). Excessive consumption of FG from CSM has been proven to negatively impact poultry growth and physiological metabolism, resulting in reduced performance and increased mortality rates [4,5,6]. The maximum allowable concentration of FG in broiler diets is estimated to be around 200 mg/kg [7].

Many techniques, such as soaking, autoclaving, microwaving, roasting, irradiation, and fermentation, can reduce or eliminate anti-nutritional compounds in feed ingredients. However, some of these methods may degrade the feedstuff’s protein nutritional value and also increase costs [1]. Among these techniques, irradiation and solid-state fermentation have recently attracted increasing attention for their ability to improve the nutritional properties, digestibility, and safety of feed [8,9]. This study aims to investigate and compare the effects of these two methods on the nutritional quality of CSM and assess the influence of each process on the performance and physiological characteristics of broiler chickens when replacing SBM with them. Solid-state fermentation involves the growth of microorganisms on substrates with limited water content. The metabolic activities of microorganisms during this process break down anti-nutritional factors and biopolymers in the raw materials, producing fermented products enriched with probiotics, organic acids, enzymes, antioxidants, and vitamins [3,10,11]. The key to a successful fermentation process is the selection of appropriate bacterial and fungal strains. *Bacillus subtilis* is a widely used probiotic in broiler diets, particularly favored in solid-state fermentation for its ability to thrive in low-moisture conditions. This bacterium can produce various enzymes, including proteases, lipases, amylases, and carboxypeptidases, and enhances the antioxidant and antimicrobial properties of the substrate during fermentation [11]. Lactic acid bacteria (LAB), known as biosafe species, can produce organic acids—primarily lactic acid—during fermentation, which lowers the substrate’s pH, inhibits harmful microorganisms, and stabilizes the fermentation process [12]. Previous evidence has shown a significant reduction in FG content in fermented CSM (FCSM) with *Bacillus subtilis*, dropping from 820 to 210 mg/kg [13]. Our recent study also indicated that fermentation of CSM with *Saccharomyces cerevisiae*, *Bacillus subtilis*, and *Lactobacillus plantarum* reduces FG content from 985 to 107 mg/kg [14]. In vivo research has shown that feeding broilers diets containing FCSM improves body weight gain and feed conversion ratio compared to diets containing CSM [4,13]. Fermented feed also plays a crucial role in improving intestinal structure and modifying gut microbiota in poultry [10]. Therefore, feeding fermented feeds can be advantageous for poultry performance and health. Radiation is a safe and reliable method for improving the nutritional quality of feedstuff [9]. The advantages of utilizing ionizing radiation such as electron beams and gamma rays in feed processing include mitigating indigestible reactions like the Maillard reaction, minimizing nutrient degradation, especially proteins, eliminating fungal and bacterial contamination, reducing anti-nutritional compounds, and simultaneously enhancing nutrient digestibility [15]. Electron beam and gamma ray irradiation, at a dose of 30 kGy, reduced gossypol and crude fiber while increasing the protein digestibility of CSM [16]. Nayefi et al. [17] found that electron beam irradiation of CSM at the dose of 30 kGy effectively reduces FG content, leading to improved weight gain and FCR when included in broiler diets compared to non-irradiated CSM. Moreover, it has been reported that including irradiated canola meal in broiler breeder diets improves protein digestibility [18]. However, to our knowledge, limited research has been conducted on replacing SBM with irradiated CSM (ICSM) in broiler diets. Nevertheless, various studies have demonstrated the positive effects of the irradiation technique on improving feed quality, presenting promising prospects for application of this method in the feed and poultry industries. Therefore, given the challenges faced by the poultry industry in sourcing SBM, this study evaluated the nutritional value of FCSM and ICSM by assessing their effects on the growth performance and physiological responses of broiler chickens when used as substitutes for SBM in their diets. The findings are expected to improve the sustainability and resilience of poultry farming while reducing chicken meat production costs.

## 2. Materials and Methods

The experimental procedures of this study were approved by the Animal Ethics Committee of the Animal Science Faculty at the University of Mohaghegh Ardabili, Ardabil, Iran, under protocol #99/185/270 FA.

### 2.1. Fermentation of CSM

The CSM used in this study was sourced from Vahdat Company (Tehran, Iran), which utilized the expander/solvent (hexane) extraction method for oil extraction. The fermentation process of CSM was conducted using *Bacillus subtilis* (PTCC1156) and *Lactobacillus plantarum* (PTCC1058), obtained from the Persian Culture Collection of the Iranian Science and Technology Research Organization in Tehran, Iran. For each kilogram of CSM, a microbial suspension was prepared by combining 1.2 L of distilled water with approximately 10^5^ CFU/mL of each bacterium. This suspension was then used to inoculate the CSM, which was thoroughly mixed and incubated for seven days at 30 °C in specialized tanks equipped with a one-way valve to release fermentation gases. Post-fermentation, the FCSM was spread on a polyethylene sheet in a room at 30 to 40 °C and dried for 3 days to approximately 900 g/kg dry matter. The dried FCSM was then ground to pass through a 0.5 mm sieve and stored at room temperature until mixed into the experimental diets. Table 1 details the alterations in pH and chemical composition of CSM resulting from the fermentation process.

### 2.2. Irradiation of CSM

The CSM was packed in polyethylene bags and subjected to electron beam irradiation at the Yazd Radiation Processing Centre (AEOI, Yazd Centre, Iran). The irradiation dose was set at 30 kGy, with a beam energy of 10 MeV, at room temperature, using a Rhodotron accelerator (model TT200, Ion Beam Applications (IBA), Ottignies-Louvain-la-Neuve, Belgium). The dose rate was 180 kGy/min, as determined by cellulose triacetate film (ISO/ASTM 51650, 2013 [19]). The uncertainty for electron radiation was 5%, and the dose uniformity ratio (Dmax/Dmin) was 1.10 [17]. Changes in the chemical composition of CSM post-irradiation are outlined in Table 1.

### 2.3. Chemical Analysis

All the chemicals used in this experiment were of analytical grade (Merck, Darmstadt, Germany). The chemical composition of the CSM, ICSM, and FCSM was analyzed according to AOAC [20] methods, which included determining dry matter (930.15), crude protein (984.13), ether extract (920.39), ash (942.05), and crude fiber (978.10). The FG content was assessed using AOCS [21] protocols with high-performance liquid chromatography. The amino acid composition of the samples was measured using the Hitachi L-8900 automated analyzer (Hitachi High-Technologies Corporation, Tokyo, Japan), which employs ion-exchange chromatography and post-column ninhydrin derivatization [22]. Samples were hydrolyzed in 6 M HCl at 110 °C for 24 h [23]. Sulphur-containing amino acids were oxidized with performic acid before hydrolysis [24]. The metabolizable energy value of CSM, FCSM, and ICSM was calculated based on their chemical composition using the following formula: AME = 21.26DM + 47.13EE − 30.85CF [25]. To determine the population of LAB, 1 g of CSM, ICSM, or FCSM was used to make serial 10-fold dilutions using buffered peptone water. Then, 0.1 mL of appropriate dilutions were spread on the plates containing modified de Man, Rogosa, and Sharpe (MRS) agar. Plates were incubated in anaerobic conditions for 24 h at 37 °C. After counting the colonies in each plate, the obtained number was multiplied by reversed dilution and reported as log10 CUF per 1 g sample. To determine the pH value, 20 g of each sample was transferred to a 250 mL beaker, and 200 mL of distilled water was added. The pH was measured using a portable pH meter (NWKbinar pH, K-21, Landsberg, Germany). Each sample was analyzed in quadruplicate for accuracy.

### 2.4. Experimental Birds, Diets, and Management

Three hundred and eighty-four healthy, one-old-day male Ross 308 broiler chickens were purchased from a commercial hatchery. Upon arrival, the chicks were weighed and randomly assigned to four experimental diets, each comprising eight replicate pens of 12 chicks, following a completely randomized design. The chicks were housed in floored cages with wood shaving in an environmentally controlled poultry shed. They had ad libitum access to fresh water and feed through the 42-day trial period. The shed’s temperature was maintained at 34 °C for the first three days, then reduced by 3 °C each week until it reached 23 °C. This temperature was kept steady until the end of the experiment. From days 1 to 7, the chicks were under continuous lighting, receiving 24 h of light daily. After the first week, the lighting schedule was adjusted to 18 h of light and 6 h of darkness per day. The experimental diets included a control diet based on corn–SBM and three other diets. In these test diets, 50% of the SBM from the control diet was replaced with either CSM, ICSM, or FCSM. All birds received the test diets in three phases: a starter diet from days 1 to 10, a grower diet from days 11 to 24, and a finisher diet from days 25 to 42 (Table 2, Table 3 and Table 4). The experimental diets were formulated according to the requirements recommended by the Ross 308 Nutrient Specifications and provided in mashed form. During the final phase of the experiment (from days 32 to 42), chromic oxide (3.0 g/kg) was added to the diets as an indigestible marker to determine the AID of nutrients.

### 2.5. Broiler Performance, Sampling, and Measurements

On days 10, 24, and 42, the body weight and feed intake for each cage were recorded. Using these data, the average daily gain (ADG) and average daily feed intake (ADFI) per bird were calculated for the total grow-out period. The feed conversion ratio (FCR) was then determined by dividing the ADFI by ADG. Mortality was monitored daily across all groups, and performance data were adjusted accordingly.

At the end of the experiment, two birds per pen were randomly selected, weighed, and euthanized via cervical dislocation to assess gastrointestinal tract (GIT) pH, ceca microflora population, and digestive enzyme activity.

Upon excision of the digestive tract, digesta samples from various sections—including the crop, gizzard, proventriculus, duodenum, jejunum, ileum, and cecum—were meticulously collected separately. The pH of each digesta sample was promptly measured using a pH meter (Envirosensors spear tip pH probe, Singapore) by inserting it into the frontal crop, gizzard, proventriculus, duodenum, jejunum, ileum, and ceca sections.

Under sterile conditions, approximately 1 g of cecal digesta was serially diluted in 0.9% sterile saline solution. Subsequently, 100 μL of each dilution was plated onto specific agar media: MRS agar for *Lactobacillus* spp., BSM agar for *Bifidobacterium* spp., MacConkey agar for *Escherichia coli*, and TSC agar for *Clostridium* spp. The bacterial cultures of *Lactobacillus*, *Bifidobacterium*, and *Clostridium* were incubated anaerobically at 37 °C for 48–72 h, whereas the *Escherichia coli* culture was incubated aerobically at 37 °C for 24 h. The microbial populations were reported as log_10_ CUF per gram of cecal digesta.

The pancreas samples and jejunal digesta contents were diluted 4-fold and 10-fold, respectively, using the phosphate-saline buffer. The diluted samples were centrifuged at 3000× *g* for 15 min and 18,000× *g* for 20 min at 4 °C, respectively. The supernatant was stored at −70 °C until enzyme assay procedures were conducted. Amylase (a-1, 4-glucan 4-glucanohydrolase, EC 3.2.1.1) activity was determined using the method of Somogyi [26]. One amylase activity unit (1 Somogyi unit) was defined as the amount of amylase that will cause formation of reducing power equivalent to 1 mg of glucose in 30 min at 38 °C per mg of intestinal digesta protein or pancreas. The substrate used in the analysis was cornstarch. Lipase (triacylglycerol lipase, EC 3.1.1.3.) activity was assayed using the method described by Tietz and Fiereck [27]. A lipase activity unit (Sigma-Tietz units) was equal to the volume (in mL) of 0.05 M NaOH required to neutralize the fatty acid liberated during a 6 h incubation with 3 mL of lipase substrate at 38 °C per mg of intestinal digesta protein or pancreas. Olive oil was used as the substrate in this assay. Protein concentration was measured following the method of Lowry et al. [28], modified for a 96-well plate. In brief, samples were incubated with the reagent mixture, and absorbance was measured at 750 nm using a microplate reader. Bovine serum albumin was used as the standard for constructing the calibration curve.

On day 42, two birds per replicate were euthanized by cervical dislocation. Following the methodology outlined by Ravindran et al. [29], the ileal digesta was collected from the section of the small intestine extending from Meckel’s diverticulum to approximately 40 mm before the ileocaecal junction. The pooled digesta collected from birds within each pen was immediately frozen at −20 °C for subsequent analysis. At the time of analysis, after drying the feed and ileal digesta samples in an oven at 65 °C for 24 h, the samples were finely ground in preparation for chemical analysis. Following this, AOAC methods [20] were used to measure the contents of dry matter, crude protein, ether extract, and ash in both processed feed and ileal samples. The chromium concentrations in both the feed and digesta samples were determined using spectrophotometry (450 nm; Spark 10 M; Tecan Group Ltd., Mannedrof, Switzerland) following wet-ash digestion with perchloric and nitric acid, in accordance with the method described by Fenton and Fenton [30]. The AID of nutrients was subsequently calculated using the equation below.

AID = 1 − [(Nutrientdigesta × Markerdiet)/(Nutrientdiet × Markerdigesta)]

At the termination of the experiment, fresh excreta samples from each pen were collected to assess concentrations of ammonia, hydrogen sulfide, and total mercaptan. The samples were placed in 3 L plastic boxes and allowed to ferment for 5 days at room temperature. Gas analysis post-fermentation was performed using a Gastec model GV-100S gas sampling pump kit [31].

### 2.6. Statistical Analyses

The data were subjected to analysis of variance (ANOVA) for a completely randomized design using GLM procedures in SAS software (Version 9.3). The Shapiro–Wilk and Levene tests were applied to assess the normality and homogeneity of variances in the data. Tukey’s post-hoc analysis was used for mean separation, with significance at *p* < 0.05.

## 3. Results

### 3.1. Growth Performance

Table 5 outlines the impact of the experimental diets on the growth performance of broiler chickens. Chickens fed diets containing untreated CSM exhibited lower ADG and ADFI and a higher FCR throughout the entire rearing period than those on other experimental diets (*p* < 0.05). Consequently, substituting 50% of SBM with untreated CSM in broiler diets reduced body weight on days 24 and 42 (*p* < 0.05). Compared to the CSM diet, birds receiving the ICSM exhibited superior ADG throughout the grow-out period and greater body weight on days 24 and 42 (*p* < 0.05). Replacing 50% of SBM with FCSM more effectively improved ADG, ADFI, and FCR compared to diets containing untreated CSM and ICSM (*p* < 0.05). No significant differences in performance parameters were observed between birds fed FCSM and those on control diets (*p* > 0.05). On days 24 and 42, birds fed the control and FCSM diets exhibited higher body weight than those on the untreated CSM and ICSM diets (*p* < 0.05).

### 3.2. Gastrointestinal pH

As shown in Table 6, including FCSM in the birds’ diet significantly decreased the pH values in the crop, ileum, and ceca compared to the other experimental groups (*p* < 0.05); however, the FCSM diet had no significant effect on the pH values of the gizzard, duodenum, and jejunum (*p* > 0.05). The other experimental diets had no impact on pH values in any of the GIT segments (*p* < 0.05).

### 3.3. Ceca Microbiota

The data presented in Table 7 depict the effect of experimental diets on the microbial population in the ceca of broiler chickens. The population of *Lactobacillus* spp. in the ceca of the group fed FCSM diets was significantly higher than the group fed other diets (*p* < 0.05). No significant differences were observed among the experimental diets in the population of *Bifidobacterium* spp. in the ceca (*p* > 0.05). Chickens fed the FCSM diets showed reduced counts of *Escherichia coli* and *Clostridium* spp. in the ceca compared to the other treatment groups (*p* < 0.05).

### 3.4. Digestive Enzyme Activity

The data in Table 8 illustrate broiler chickens’ digestive enzyme activities in the jejunum and pancreas in response to the dietary treatments. The data showed that replacing 50% of SBM with FCSM in the broilers’ diet increased amylase and protease activity in the jejunum (*p* < 0.05). However, no significant changes between experimental groups were observed in amylase, protease, and lipase activity in the pancreas and lipase activity in the jejunum (*p* > 0.05).

### 3.5. Apparent Ileal Digestibility of Nutrients

Table 9 details the effects of experimental diets on the AID of nutrients in broiler chickens. Substituting 50% of SBM with FCSM in the broiler diets improved the AID of crude protein and ether extract compared to diets containing untreated CSM and ICSM (*p* < 0.05); however, no significant differences were recorded between the control diet and the FCSM-containing diet (*p* > 0.05). Feeding broilers with ICSM enhanced the AID of crude protein compared to the untreated CSM-containing diet (*p* < 0.05); however, crude protein digestibility in the ICSM group was lower than in the FCSM and control diets (*p* < 0.05).

### 3.6. Excreta Gas Emissions

Table 10 shows the concentrations of excreta gas emissions—ammonia, hydrogen sulfide, and total mercaptan—in broiler chickens under different dietary treatments. Broiler chickens fed diets containing FCSM exhibited reduced ammonia emissions compared to those on other dietary treatments (*p* < 0.05). However, the experimental diets did not influence hydrogen sulfide emissions and total mercaptan (*p* > 0.05). 

## 4. Discussion

The utilization of CSM in monogastric animal diets, particularly poultry, is primarily limited due to the presence of FG, which adversely affects protein digestibility, digestive enzyme activity such as pepsinogen, pepsin and trypsin, and overall growth performance in birds [7]. Our study aligns with prior findings [5,17], which indicated that replacing SBM with CSM resulted in reduced ADFI and ADG and increased FCR, and compromised productive performance. This limitation underscores the necessity for effective strategies to mitigate FG’s negative impacts in CSM for optimal poultry nutrition. To address this, we investigated the effects of CSM processed through microbial fermentation and electron beam irradiation techniques on the productive performance and physiological response of broiler chickens in our study. Replacing 50% of SBM with FCSM in the diet improved the body weight, ADG, and FCR of broiler chickens compared to the ICSM and CSM treatment groups. In this study, FG values for CSM, ICSM, and FCSM were 932, 494, and 100 mg/kg, respectively. Therefore, the superior performance of chickens in the FCSM group can primarily be attributed to its lower FG values than the other two groups. Birds fed diets containing FCSM showed no significant differences in body weight, ADG, or FCR compared to those fed the control diet. Tang et al. [13] found that replacing 8% of SBM with FCSM in broiler diets improved ADG compared to the control diet. Our previous study also revealed that use of 10% and 20% FCSM instead of SBM in broiler diets improved the body weight gain and FCR compared to diets containing CSM [4]. Fermented feedstuffs such as rapeseed meal [32], SBM [33], and palm kernel cake [34] also enhanced feed efficiency in broiler chickens. A literature review indicates that improvement in the growth performance of birds fed diets containing fermented plant protein sources is linked to the reduction of anti-nutritional substances, an increase in nutrient quality, and the enhanced populations of beneficial bacteria improving GIT health and functionality in poultry [32,35,36]. The findings of this study also demonstrate that including FCSM in the diet positively affects the gut pH, ceca microbial population, digestive enzyme activity, and nutrient digestibility, in which these improvements can lead to enhanced performance in broiler chickens. Consequently, FCSM can replace up to 50% of SBM in broiler diets without adversely affecting production performance. On the other hand, the data from the ICSM group during the entire grow-out period indicated that birds fed diets containing ICSM (30 kGy) had superior body weight and ADG compared to those on a CSM diet. However, growth performance in the ICSM group was significantly lower than in the FCSM and control diets. Consistent with these findings, Nayefi et al. reported that broiler chickens fed diets containing 12% ICSM (30 kGy) exhibited higher body weight gain and feed intake than those on a 12% CSM diet [17]. These authors attributed the improved performance to reduced FG content and increased nutrient digestibility by irradiation. Likewise, in this study, a significant decrease in FG content and an increase in crude protein digestibility were observed in the ICSM group compared to the CSM group. Other studies have demonstrated that using irradiated feed enhances the growth performance and ileal digestibility of broiler chickens compared to raw feed [37]. Electron beam irradiation can induce the formation of bonds between gossypol molecules (aggregation), interactions with other molecules (cross-linking), and the fragmentation and breakdown of gossypol [16,17].

Maintaining the proper pH in the GIT is critical for optimal digestive enzyme function and maintaining a balanced gut microflora. Stabilizing the GIT microbiota plays a pivotal role in intestinal health and function and efficient nutrient absorption [38]. The outcomes of the present study showed that feeding FCSM decreased the pH values in the crop, ileum, and ceca contents compared to the other experimental diets. Consistent with these findings, previous studies have shown that including fermented products in the diet of broiler chickens [32,39] and laying hens [14,40] can shift the balance of gut microbiota to favor beneficial bacteria and reduce pH throughout the GIT. In addition to improving the nutritional properties of feed through microbial fermentation, fermented feed features low pH, high counts of lactobacilli, and high concentrations of organic acids, like lactic acid [41,42]. Feeding birds fermented feed creates an acidic environment in the upper digestive tract, promoting the growth of lactobacilli. This increased *lactobacillus* presence boosts the production of lactic acid and short-chain fatty acids, thereby lowering the pH of digestive tract biology, creating a suitable environment for optimal digestive function [12,41]. This also establishes a competitive platform that helps form natural defense barriers against infections and pathogens like *Escherichia coli*, bolstering overall bird health [35]. In this study, birds fed FCSM diets exhibited significantly higher populations of *Lactobacillus* in the ceca compared to birds on other diets. Furthermore, feeding birds FCSM diets reduced the counts of *Escherichia coli* and *Clostridium* in the ceca compared to the other treatment groups. A similar observation was seen in the experiment of Jazi et al. [4]. They found that broiler chickens fed FCSM had a higher LAB count and lower coliform in the ileum. Furthermore, Elbaz et al. [43] showed that the dietary inclusion of fermented rapeseed meal stabilizes the gut microbiota, thereby reducing the colonization of *Escherichia coli* in the ceca.

The function of digestive enzyme activity is closely related to the intestinal digestion and absorption of nutrients [44]. These enzymes, including amylase, protease, and lipase, catalyze the breakdown of complex nutrients into simpler forms that can be readily absorbed by the intestinal lining. This enzymatic activity is critical for optimizing nutrient utilization in domestic animals, playing a key role in metabolic processes and overall animal performance. Although numerous studies have shown that fermented feed/ingredients improve the growth performance of birds [10,32,45,46], there is limited research on how they influence gut function, including digestive enzyme activities and nutrient digestibility. Interestingly, the current data indicate that substituting SBM with FCSM in broilers’ diets increased amylase and protease activity in the jejunum. Sun et al. [47] have reported similar findings, indicating that including FCSM in broiler chickens’ diets increased the activities of intestinal amylase, protease, and lipase. They noted that viable LAB and Bacillus bacteria in FCSM could enhance enzyme activity by producing protease and amylase enzymes [47]. A recent study also showed that feeding broilers fermented rapeseed meal increases intestinal amylase and lipase activities and enhances the villus height in the ileum [43]. Based on the literature and observations from the current study, enhancing digestive enzyme activity in the FCSM group can be attributed to modifying the intestinal microbiota ecosystem and improving the intestinal lining’s integrity [41,46,47]. Moreover, the presence of organic acids in fermented feed facilitates the release of secretin and free protons, lowering digesta pH. This acidic environment promotes the secretion of enzymes in the gut, thereby enhancing the efficiency of nutrient digestion and absorption [48,49].

Quantitative evaluation of nutrient digestibility offers insights into the nutritional and physiological processes related to digestion capacity and GIT functionality. In this study, replacing SBM with FCSM in the diet improved the AID of crude protein and ether extract compared to diets containing CSM and ICSM. Alshelmani et al. [34] observed a significant improvement in the AID of crude protein and amino acids in fermented palm kernel cake compared to raw palm kernel cake. This effect might be due to microorganisms’ metabolic activity during fermentation, which secretes enzymes like cellulase, phytase, and xylanase. These enzymes help break down fiber materials into simpler sugars like monosaccharides [35,41]. The inclusion of fermented SBM in the diet of broiler chickens increased crude protein digestibility compared to the control group [50]. Moreover, other studies have shown that the fermentation of canola meal improved the digestibility of crude protein in broiler chickens compared to raw canola meal [51]. This enhancement was attributed to the breakdown of protein and the formation of peptides and amino acids, increasing the protein’s availability and solubility [35,41,52]. It is worth noting that fermented feeds have beneficial effects on microbial ecosystems, intestinal structure, integrity, digestive enzyme activity, and immunity, which could lead to better digestion and absorption capacity in poultry. In the present study, feeding ICSM improved the AID of crude protein compared to the CSM diet. This improvement could be attributed to the reduction of anti-nutritional factors such as FG. Bahraini et al. found that treating CSM with electron beam irradiation at a dose of 30 kGy reduced FG and crude fiber, while increasing protein digestibility in Leghorn cockerels [16]. Irradiation appears to have caused protein denaturation, exposing hydropholic amino acids, particularly aromatic ones that play pivotal roles at the active sites of pepsin and trypsin enzymes. Chamani et al. reported that use of irradiated canola meal in broiler breeder diets increases the digestibility of protein [18].

Noxious gases, including ammonia, hydrogen sulfide, and methyl mercaptan, are the main air pollutants in poultry sheds. The emission of these gases is closely related to nutrient digestibility [31]. Improved nutrient digestibility in animals allows for the complete oxidative breakdown of organic substrates in the intestine [53]. This decreases fecal odor and significantly reduces noxious gas emissions, contributing to a healthier environment and enhancing animal welfare. Ammonia production mainly results from inefficient utilization of dietary protein, which increases nitrogen excretion in the form of uric acid [54]. In the present study, feeding birds with diets containing FCSM improved the balance of the microbiota ecosystem in the ceca and increased nutrient digestibility compared to the other diet. Therefore, the reduction in excreta ammonia levels observed in this study may be linked to an increase in the *Lactobacillus* population in the ceca and improved CP digestibility [53,54]. The results of the present study align with previous studies [49], which demonstrated that including fermented feed in broiler diet reduced ammonia gas emissions from excreta.

## 5. Conclusions

As demonstrated, microbial fermentation of CSM is a more effective approach than irradiation for enhancing its nutritional value. Additionally, the functional ingredients in fermented feed can positively impact animal gut health and functionality. The current study showed that replacing 50% of SBM with FCSM enhanced growth performance by improving cecal microbiota balance, nutrient digestibility, and digestive enzyme activity in broiler chickens compared to untreated CSM and ICSM. This study indicated that FCSM can replace up to 50% of SBM in broiler diets without adversely impacting production performance and overall health. Therefore, this processed protein source can serve as a suitable alternative to SBM in broiler chicken diets. Future research should focus on optimizing the fermentation process to specifically target and mitigate all anti-nutritional factors in these protein sources, while also standardizing their nutritional value. This approach could undoubtedly create new opportunities for the poultry feed industry. Furthermore, additional research is needed to investigate the long-term effects of FCSM on gut health, the immune system, and production sustainability under industrial conditions. Such studies could reduce reliance on SBM, improve environmental sustainability, and increase the economic efficiency of poultry production.

## Figures and Tables

**Table 1:** The chemical composition of untreated, fermented, and irradiated cottonseed meal.

Item	CSM *	FCSM *	ICSM *	SEM	*p*-Value
pH	5.50 ^a^	3.90 ^b^	5.23 ^a^	0.30	0.006
Lactic acid bacteria (log_10_ cfu/g)	4.87 ^b^	10.03 ^a^	2.15 ^c^	0.05	<0.001
Crude protein (%)	37.54 ^b^	40.36 ^a^	37.65 ^b^	0.70	0.015
Dry matter (%)	92.90	91.45	92.10	0.84	0.210
Crude fiber (%)	11.78 ^a^	9.72 ^b^	11.20 ^a^	0.09	0.027
Ether extract (%)	1.45	1.01	1.27	0.04	0.431
Ash (%)	6.49	6.73	6.60	0.33	0.690
Free gossypol (mg/kg)	932 ^a^	100 ^c^	494 ^b^	7.05	<0.001
Indispensable amino acids (%)					
Arginine	4.10	4.31	4.26	0.80	0.200
Histidine	1.09	1.20	1.10	0.45	0.342
Isoleucine	1.18	1.27	1.22	0.25	0.290
Leucine	2.21	2.29	2.18	0.50	0.463
Lysine	1.43	1.59	1.40	0.61	0.557
Methionine	0.52	0.61	0.55	0.65	0.721
Phenylalanine	2.10	2.28	2.12	0.98	0.276
Threonine	1.23	1.40	1.25	0.71	0.605
Valine	1.80	1.88	1.79	0.59	0.764
Dispensable amino acids (%)					
Alanine	1.48	1.57	1.50	1.10	0.420
Asparagine	3.49	3.50	3.39	1.02	0.161
Cysteine	0.55	0.59	0.51	0.41	0.597
Glutamine	8.72	9.01	8.95	1.90	0.233
Glycine	1.55	1.60	1.60	0.81	0.600
Proline	1.40	1.52	1.45	0.56	0.450
Serine	1.67	1.80	1.69	0.43	0.422
Tyrosine	0.87	0.88	0.80	0.78	0.301

* CSM = untreated cottonseed meal; FCSM = fermented cottonseed meal; ICSM = irradiated cottonseed meal. ^a–c^ Means with a common superscript in each row are not significantly different (*p* < 0.05).

**Table 2:** Ingredients and calculated nutrient composition of the experimental diets for the starter phase (days 1–10).

	Experimental Diets			
Ingredients (%)	CON	CSM	FCSM	ICSM
Corn	54.18	52.77	54.28	53.18
Soybean meal	38.10	17.81	16.57	17.49
Cottonseed meal (CSM)	0	19.05	0	0
Fermented cottonseed meal (FCSM)	0	0	19.05	0
Irradiated cottonseed meal (ICSM)	0	0	0	19.05
Vegetable oil	3.06	4.61	4.34	4.52
Carbonate calcium	0.79	0.94	0.95	0.94
Di-calcium phosphate	2.16	2.09	2.09	2.09
Salt	0.27	0.27	0.27	0.27
Sodium bicarbonate	0.14	0.13	0.13	0.13
Vitamin premix ^1^	0.25	0.25	0.25	0.25
Mineral premix ^2^	0.25	0.25	0.25	0.25
DL-Methionine	0.35	0.42	0.41	0.42
L- Lysine	0.10	0.68	0.69	0.68
L-Threonine	0.24	0.26	0.25	0.26
L-Valine	0.11	0.21	0.21	0.21
L-Isoleucine	0	0.26	0.26	0.26
Total	100	100	100	100
Calculated composition				
Metabolizable energy (Kcal/kg)	2950	2950	2950	2950
Crude protein (%)	21.53	20.38	20.49	20.30
SID Lysine (%)	1.23	1.23	1.23	1.23
SID Methionine (%)	0.63	0.63	0.63	0.63
SID Methionine + Cysteine (%)	0.91	0.91	0.91	0.91
SID Threonine (%)	0.80	0.80	0.80	0.80
Calcium (%)	0.96	0.96	0.96	0.96
Available phosphorous (%)	0.48	0.48	0.48	0.48
Sodium (%)	0.16	0.16	0.16	0.16
Free gossypol (mg/kg)	-	177.54	19.05	94.11

^1^ Supplied per kg of diet: 1.8 mg all-trans-retinyl acetate; 0.02 mg cholecalciferol; 8.3 mg alphatocopheryl acetate; 2.2 mg menadione; 2 mg pyridoxine HCl; 8 mg cyanocobalamin; 10 mg nicotine amid; 0.3 mg folic acid; 20 mg D-biotin; 160 mg choline chloride. ^2^ Supplied per kg of diet: 32 mg Mn (MnSO_4__H_2_O); 16 mg Fe (FeSO_4__7H_2_O); 24 mg Zn (ZnO); 2 mg Cu (CuSO_4__5H_2_O); 800 µg I (KI); 200 µg Co (CoSO_4_); 60 µg Se (NaSeO_3_).

**Table 3:** Ingredients and calculated nutrient composition of the experimental diets for the grower phase (days 11–24).

	Experimental Diets			
Ingredients (%)	CON	CSM	FCSM	ICSM
Corn	56.19	55.26	56.62	55.60
Soybean meal	35.40	16.20	15.05	15.91
Cottonseed meal (CSM)	0	17.70	0	0
Fermented cottonseed meal (FCSM)	0	0	17.70	0
Irradiated cottonseed meal (ICSM)	0	0	0	17.70
Vegetable oil	4.12	5.51	5.26	5.43
Carbonate calcium	0.71	0.86	0.87	0.86
Di-calcium phosphate	1.92	1.85	1.86	1.86
Salt	0.27	0.27	0.27	0.27
Sodium bicarbonate	0.14	0.13	0.14	0.14
Vitamin premix ^1^	0.25	0.25	0.25	0.25
Mineral premix ^2^	0.25	0.25	0.25	0.25
DL-Methionine	0.33	0.40	0.40	0.41
L- Lysine	0.22	0.63	0.64	0.63
L-Threonine	0.11	0.25	0.24	0.25
L-Valine	0.09	0.20	0.20	0.20
L-Isoleucine	0	0.26	0.26	0.26
Total	100	100	100	100
Calculated composition				
Metabolizable energy (Kcal/kg)	3050	3050	3050	3050
Crude protein (%)	20.43	19.27	19.37	19.20
SID Lysine (%)	1.15	1.15	1.15	1.15
SID Methionine (%)	0.61	0.61	0.61	0.61
SID Methionine + Cysteine (%)	0.87	0.87	0.87	0.87
SID Threonine (%)	0.76	0.76	0.76	0.76
Calcium (%)	0.87	0.87	0.87	0.87
Available phosphorous (%)	0.435	0.435	0.435	0.435
Sodium (%)	0.16	0.16	0.16	0.16
Free gossypol (mg/kg)	-	165.0	17.70	87.44

^1^ Supplied per kg of diet: 1.8 mg all-trans-retinyl acetate; 0.02 mg cholecalciferol; 8.3 mg alphatocopheryl acetate; 2.2 mg menadione; 2 mg pyridoxine HCl; 8 mg cyanocobalamin; 10 mg nicotine amid; 0.3 mg folic acid; 20 mg D-biotin; 160 mg choline chloride. ^2^ Supplied per kg of diet: 32 mg Mn (MnSO_4__H_2_O); 16 mg Fe (FeSO_4__7H_2_O); 24 mg Zn (ZnO); 2 mg Cu (CuSO_4__5H_2_O); 800 µg I (KI); 200 µg Co (CoSO_4_); 60 µg Se (NaSeO_3_).

**Table 4:** Ingredients and calculated nutrient composition of the experimental diets for the finisher phase (days 25–42).

	Experimental Diets			
Ingredients (%)	CON	CSM	FCSM	ICSM
Corn	60.26	59.70	60.90	60.01
Soybean meal	30.88	13.90	16.57	17.49
Cottonseed meal (CSM)	0	15.44	0	0
Fermented cottonseed meal (FCSM)	0	0	15.44	0
Irradiated cottonseed meal (ICSM)	0	0	0	15.44
Vegetable oil	4.90	6.06	5.85	6.00
Carbonate calcium	0.66	0.79	0.80	0.79
Di-calcium phosphate	1.72	1.66	1.66	1.66
Salt	0.27	0.27	0.27	0.27
Sodium bicarbonate	0.15	0.14	0.14	0.14
Vitamin premix ^1^	0.25	0.25	0.25	0.25
Mineral premix ^2^	0.25	0.25	0.25	0.25
DL-Methionine	0.30	0.36	0.36	0.36
L- Lysine	0.20	0.56	0.57	0.57
L-Threonine	0.09	0.23	0.22	0.23
L-Valine	0.07	0.17	0.17	0.17
L-Isoleucine	0	0.22	0.22	0.22
Total	100	100	100	100
Calculated composition				
Metabolizable energy (Kcal/kg)	3150	3150	3150	3150
Crude protein (%)	18.67	17.60	17.68	17.53
SID Lysine (%)	1.03	1.03	1.03	1.03
SID Methionine (%)	0.56	0.56	0.56	0.56
SID Methionine + Cysteine (%)	0.80	0.80	0.80	0.80
SID Threonine (%)	0.80	0.80	0.80	0.80
Calcium (%)	0.79	0.79	0.79	0.79
Available phosphorous (%)	0.395	0.395	0.395	0.395
Sodium (%)	0.16	0.16	0.16	0.16
Free gossypol (mg/kg)	-	143.90	15.44	76.28

^1^ Supplied per kg of diet: 1.8 mg all-trans-retinyl acetate; 0.02 mg cholecalciferol; 8.3 mg alphatocopheryl acetate; 2.2 mg menadione; 2 mg pyridoxine HCl; 8 mg cyanocobalamin; 10 mg nicotine amid; 0.3 mg folic acid; 20 mg D-biotin; 160 mg choline chloride. ^2^ Supplied per kg of diet: 32 mg Mn (MnSO_4__H_2_O); 16 mg Fe (FeSO_4__7H_2_O); 24 mg Zn (ZnO); 2 mg Cu (CuSO_4__5H_2_O); 800 µg I (KI); 200 µg Co (CoSO_4_); 60 µg Se (NaSeO_3_).

**Table 5:** Effect of experimental diets on the growth performance parameters in broiler chickens ^1^.

	Experimental Diets ^2^					
Item	CON	CSM	FCSM	ICSM	SEM	*p*-Value
Days 1–42						
Average daily gain, g	61.1 ^a^	52.5 ^c^	60.4 ^a^	55.7 ^b^	1.34	0.001
Average daily feed intake, g	106.45 ^a^	100.01 ^b^	107.12 ^a^	104.76 ^ab^	1.50	0.004
Feed conversion ratio, g/g	1.74 ^b^	1.90 ^a^	1.77 ^b^	1.88 ^a^	0.03	0.018
Body weight, g						
Day 10	233	228	231	223	3.20	0.352
Day 24	1015 ^a^	852 ^c^	998 ^a^	907 ^b^	25.65	0.014
Day 42	2612 ^a^	2250 ^c^	2582 ^a^	2384 ^b^	32.16	0.001

^1^ Values are the means of eight replicate pens of 12 chicks. ^2^ CON = control (basal diet); CSM = untreated cottonseed meal; FCSM = fermented cottonseed meal; ICSM = irradiated cottonseed meal. ^a–c^ Means with a common superscript in each row are not significantly different (*p* < 0.05).

**Table 6:** Effect of experimental diets on the gastrointestinal tract pH value in broiler chickens.

	Experimental Diets ^1^					
Item	CON	CSM	FCSM	ICSM	SEM	*p*-Value
Crop	4.85 ^a^	4.79 ^a^	4.42 ^b^	4.82 ^a^	0.06	<0.001
Gizzard	2.76	2.70	2.57	2.69	0.10	0.118
Proventriculus	5.30	5.34	5.19	5.28	0.12	0.196
Duodenum	5.92	5.96	5.82	5.94	0.06	0.413
Jejunum	6.09	6.01	5.90	6.05	0.10	0.560
Ileum	5.74 ^a^	5.71 ^a^	5.32 ^b^	5.68 ^a^	0.05	<0.001
Ceca	5.61 ^a^	5.65 ^a^	5.21 ^b^	5.59 ^a^	0.07	0.001

^1^ CON = control (basal diet); CSM = untreated cottonseed meal; FCSM = fermented cottonseed meal; ICSM = irradiated cottonseed meal. ^a,b^ Means with a common superscript in each row are not significantly different (*p* < 0.05).

**Table 7:** Effect of experimental diets on the ceca microbial population in broiler chickens.

	Experimental Diets ^1^					
Item, log_10_ cfu/g	CON	CSM	FCSM	ICSM	SEM	*p*-Value
*Bifidobacterium* spp.	8.10	8.23	8.49	8.12	0.45	0.125
*Lactobacillus* spp.	8.24 ^b^	8.18 ^b^	9.20 ^a^	8.16 ^b^	0.20	<0.001
*Escherichia coli*	5.95 ^a^	5.90 ^a^	5.13 ^b^	5.87 ^a^	0.17	0.001
*Clostridium* spp.	6.03 ^a^	6.07 ^a^	5.20 ^b^	5.97 ^a^	0.14	<0.001

^1^ CON = control (basal diet); CSM = untreated cottonseed meal; FCSM = fermented cottonseed meal; ICSM = irradiated cottonseed meal. ^a,b^ Means with a common superscript in each row are not significantly different (*p* < 0.05).

**Table 8:** Effect of experimental diets on the digestive enzyme activity of pancreas and jejunum in broiler chickens (U/mg of digesta protein).

	Experimental Diets ^1^					
Item	CON	CSM	FCSM	ICSM	SEM	*p*-Value
Pancreas						
Amylase	41.80	40.10	43.72	42.50	1.39	0.380
Protease	150.53	147.71	149.08	147.24	2.80	0.242
Lipase	45.17	46.76	45.11	44.88	1.17	0.421
Jejunum						
Amylase	16.19 ^b^	15.80 ^b^	20.97 ^a^	15.31 ^b^	0.53	0.004
Protease	78.01 ^b^	77.49 ^b^	87.54 ^a^	76.50 ^b^	2.20	0.001
Lipase	20.22	21.85	22.76	20.90	1.26	0.509

^1^ CON = control (basal diet); CSM = untreated cottonseed meal; FCSM = fermented cottonseed meal; ICSM = irradiated cottonseed meal. ^a,b^ Means with a common superscript in each row are not significantly different (*p* < 0.05).

**Table 9:** Effect of experimental diets on the apparent Ileal digestibility of nutrients (%) in broiler chickens.

	Experimental Diets ^1^					
Item	CON	CSM	FCSM	ICSM	SEM	*p*-Value
Dry matter	65.46	64.22	66.97	65.15	0.61	0.690
Crude protein	74.80 ^a^	66.50 ^c^	75.01 ^a^	70.70 ^b^	0.95	0.002
Ether extract	80.97 ^a^	75.14 ^b^	79.20 ^a^	75.65 ^b^	0.87	0.001
Organic matter	65.73	63.22	65.85	64.07	1.15	0.489

^1^ CON = control (basal diet); CSM = untreated cottonseed meal; FCSM = fermented cottonseed meal; ICSM = irradiated cottonseed meal. ^a–c^ Means with a common superscript in each row are not significantly different (*p* < 0.05).

**Table 10:** Effect of experimental diets on the excreta gas emission in broiler chickens.

	Experimental Diets ^1^					
Item	CON	CSM	FCSM	ICSM	SEM	*p*-Value
Ammonia, ppm	37.15 ^a^	38.10 ^a^	32.54 ^b^	37.40 ^a^	0.40	0.001
Hydrogen sulfide, ppm	3.88	3.83	3.65	3.79	0.28	0.410
Methyl mercaptan, ppm	3.23	3.11	3.01	3.15	0.35	0.576

^1^ CON = control (basal diet); CSM = untreated cottonseed meal; FCSM = fermented cottonseed meal; ICSM = irradiated cottonseed meal. ^a,b^ Means with a common superscript in each row are not significantly different (*p* < 0.05).

## Data Availability

All available data are incorporated into the manuscript.
